# P-1002. From Risk to Prevention: Real-World Evaluation of Oral Vancomycin for Clostridioides difficile Prophylaxis

**DOI:** 10.1093/ofid/ofaf695.1199

**Published:** 2026-01-11

**Authors:** Megan Szesnat, Amanda Lefemine, Stephanie Bills

**Affiliations:** Advocate Health: Atrium Health, Charlotte, North Carolina; Advocate Health: Atrium Health Antimicrobial Support Network, Charlotte, NC; Advocate Health: Atrium Health, Charlotte, North Carolina

## Abstract

**Background:**

*Clostridioides difficile* is the leading cause of hospital-acquired infectious diarrhea worldwide. One frequently used method to combat *C. difficile* infection (CDI) is prophylaxis with oral vancomycin. However, there is a lack of consensus regarding patient selection and optimal prophylactic regimen.

**Figure 1.**
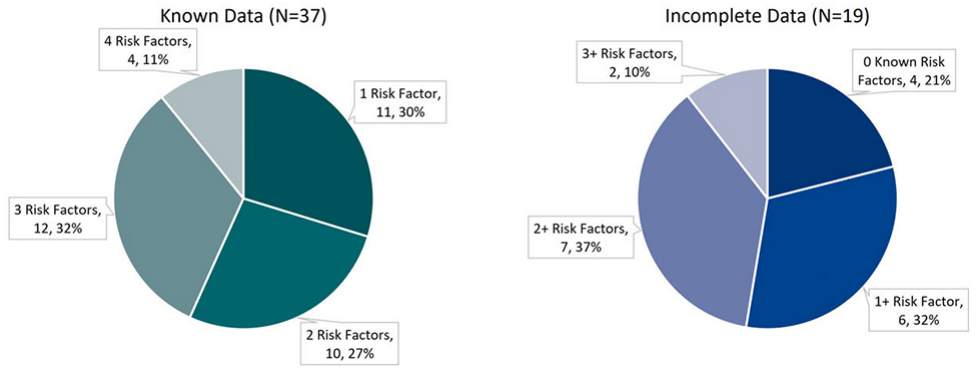
Baseline CDI Risk Factors Risk factors: age >/= 65 years, immunocompromised status, most recent CDI severe or fulminant, CDI within prior 90 days

**Figure 2.**
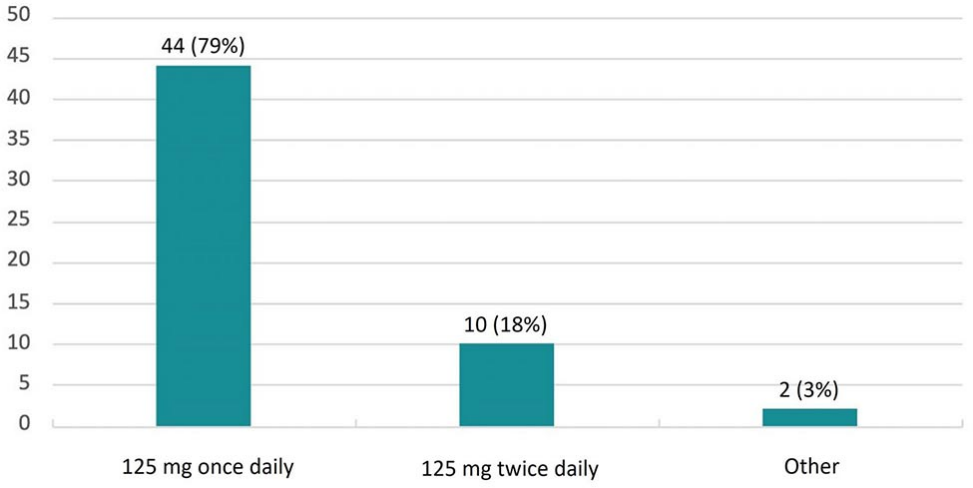
OVP Dose (N=56)

**Methods:**

This was a retrospective evaluation of adult patients admitted to Atrium Health’s Carolinas Medical Center from June 2022 through May 2024 receiving oral vancomycin prophylaxis (OVP) for at least 72 hours. Patients were excluded if they received any additional CDI targeted antibiotics during the study period. The primary objective was to describe OVP prescribing patterns. Secondary objectives included assessing baseline CDI risk factors and the incidence of 90-day CDI and vancomycin resistant enterococcus (VRE) infection. The risk factor analysis included age ≥ 65 years, immunocompromised status, severity of most recent CDI, and CDI within prior 90 days.

**Figure 3.**
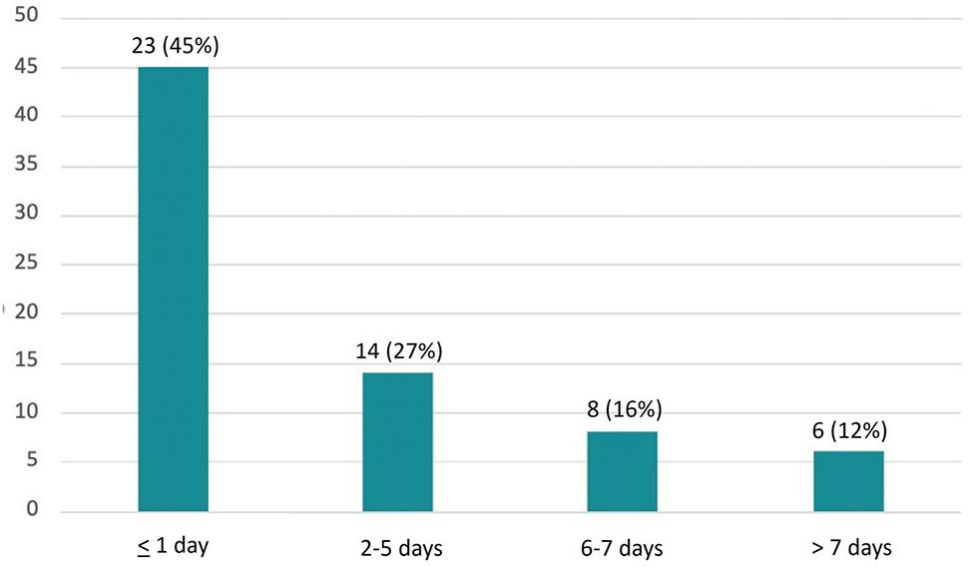
OVP Duration after Completion of Systemic Antibiotics (N=51)

**Results:**

A total of 56 patients were included in the analysis, and all but one patient had a history of CDI. Over 90% had at least one established CDI risk factor, and 63% had multiple risk factors (Figure 1), including age ≥ 65 years (46%), immunocompromised status (39%), recent severe CDI (59%), and CDI within the past 90 days (74%). However, the analysis was limited by incomplete data in 19 patients regarding their most recent CDI. Nearly all patients (95%) received concurrent systemic antibiotics, with 64% receiving at least one high CDI-risk agent. OVP was initiated based on a recommendation from infectious diseases in 79% of cases. The most common regimen was OVP 125 mg once daily (Figure 2), and OVP was discontinued within 24 hours of completion of systemic antibiotics for 45% of patients (Figure 3). Two patients developed CDI within 90 days; both received OVP 125 mg once daily that was discontinued within 24 hours of completing a high-risk antibiotic course. Additionally, two patients developed a VRE infection within 90 days.

**Conclusion:**

OVP patient selection should include an assessment of CDI risk factors. A regimen of 125 mg once daily was effective for preventing 90-day CDI in most patients. It may be beneficial to extend OVP duration past completion of systemic antibiotics to prevent additional recurrences.

**Disclosures:**

All Authors: No reported disclosures

